# Supplementary material for: Osteosarcoma Multi-Omics Landscape and Subtypes
Source: Cancers (Basel). 2023 Oct 13;15(20):4970. doi: 10.3390/cancers15204970 (PMC10605601; doi:10.3390/cancers15204970)
Supplement: Supplementary file 1 [file cancers-15-04970-s001.zip › cancers-2475060-supplementary.pdf]

Supplementary Materials: Osteosarcoma Multi-omics Landscape and Subtypes

Shan Tang [1,2], Ryan Roberts [3], Lijun Cheng [2], and Lang Li [1,2,*]

[1] College of Pharmacy, The Ohio State University, Columbus, OH 43210
[2] Department of Biomedical Informatics, College of Medicine, The Ohio State University, Columbus, OH 43210
[3] Department of Pediatrics, College of Medicine, The Ohio State University, Columbus, OH 43210
* Correspondence: Corresponding author: Lang Li, Lang.Li@osumc.edu, 1800 Cannon Drive, 240 Lincoln Tower, Columbus, OH 43210

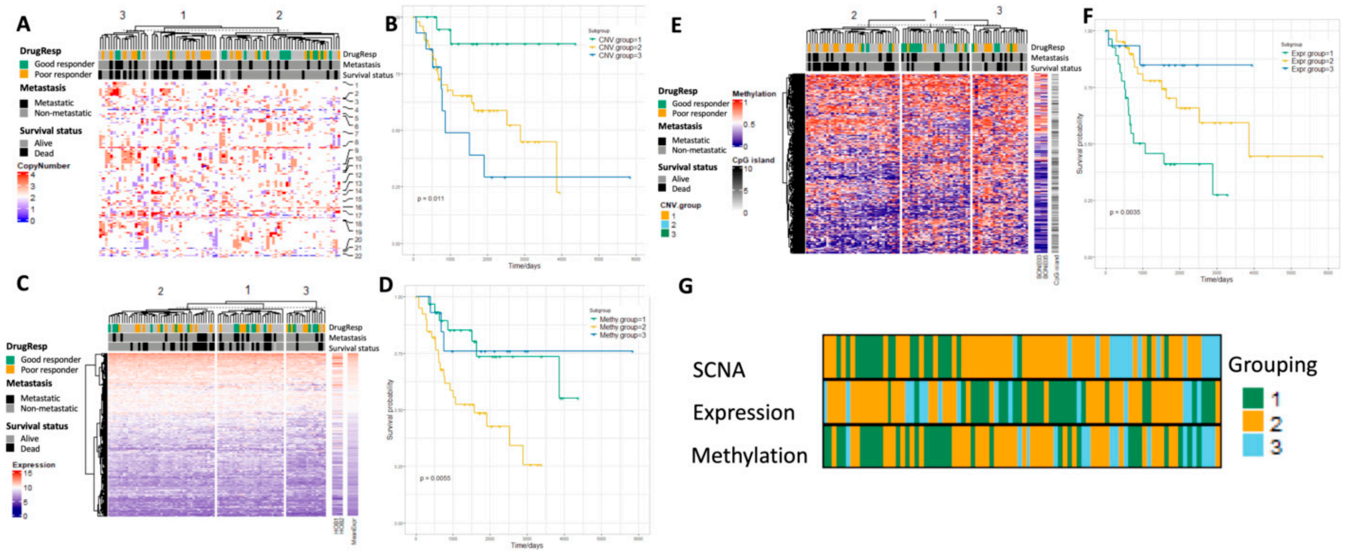

Figure S1. Unsupervised clustering of: A. SCNA, C. gene expression, E. methylation to separate 86 OS samples (validation set) into three subgroups; B, D, F: Survival plots for three subgroups based on different data sources; G. difference in subgrouping using clustering on single-platform data.

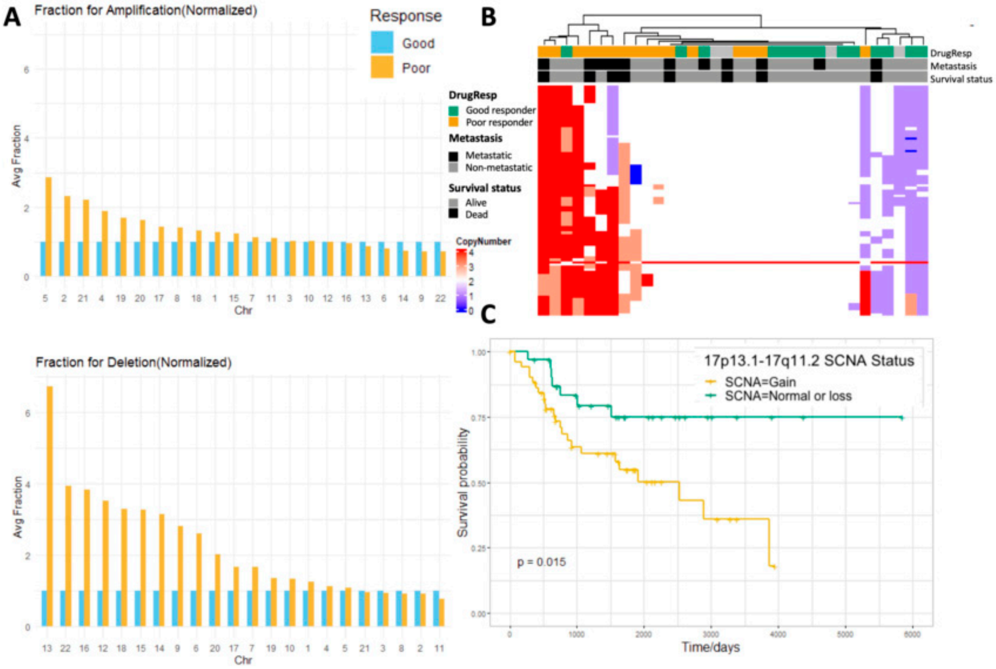

Figure S2. A. Fraction of amplified or deleted gene region across chromosome 1-22 (normalized on the data of good responders to chemo-treatment); B. SCNA profiles of 17p13.1-17q11.2 for validation set; C. survival plot based on the 17p13.1-17q11.2 SCNA status (gain vs. normal or loss).

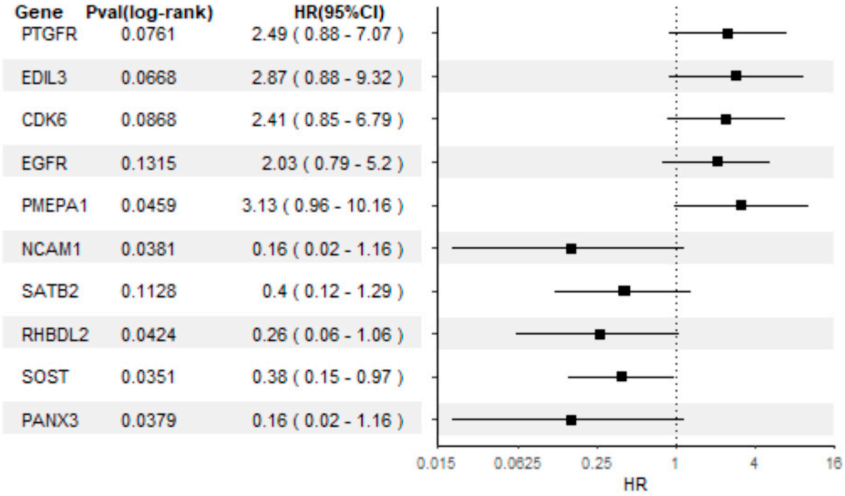

Figure S3. The effects of MRs' expression on survival outcome showed by the forest plot on validation set.

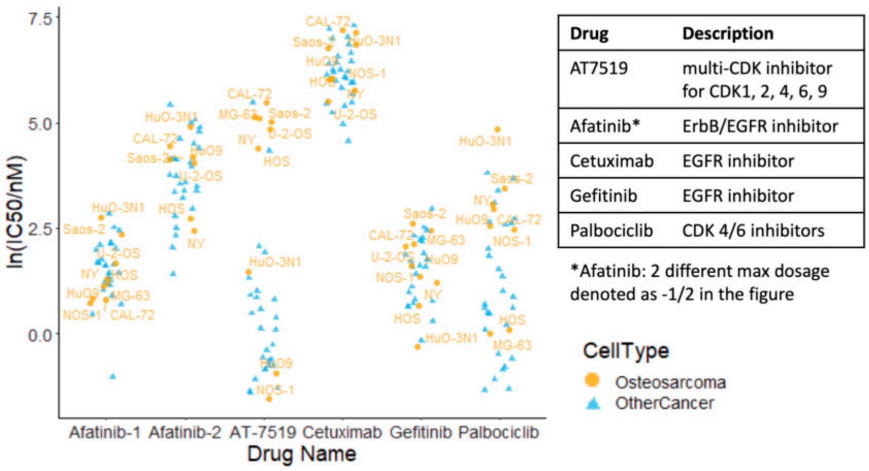

Figure S4. OS cell lines' response to CDKi and EGFRi from GDSC v17.3.

Table S1. Data source and process pipeline.

Source	Sample	Data	Platform	Process pipe line
TARGET	OS patients	SCNA	Affymetrix GW Human SNP 6.0 Array	Birdsuite (1.5.5) and PennCNV
		Expression	Affymetrix Human Exon 1.0 ST Array	R Affy, SVA package, MARINa
		Methylation	Illumina HumanMethylation450 BeadChip	Lumi
Dr. Korsching	OS patients	SCNA (E-MTAB-3034)	Affymetrix GW Human SNP 6.0 Array	Birdsuite (1.5.5) and PennCNV
		Expression (A-AFFY-142)	Affymetrix Human Exon 1.0 ST Array	R Affy, SVA package, MARINa
GSE125645	Bone samples	Methylation	Illumina HumanMethylation450 BeadChip	Lumi

Table S2. Individual genes with significant change in SCNA, expression and methylation among the three subgroups.

Table S3. 21 genes that have p-values smaller than 0.01 in SCNA, gene expression and methylation from training and validation sets.

GENE	CHR	GAIN%	LOSS%	SCNA_T	SCNA_V	EXPR_T	EXPR_V	METHY
**ARNT**	1	0.00	69.77	3.33E-03	4.30E-02	1.35E-05	2.47E-02	1.15E-04
**NADK**	1	11.63	6.98	3.19E-05	5.97E-02	2.25E-03	4.28E-02	1.08E-04
**TMEM151B**	6	20.93	2.33	3.02E-04	1.26E-02	2.85E-05	4.54E-02	4.29E-04
**MLLT3**	9	19.77	10.47	3.84E-03	4.87E-02	2.76E-03	3.60E-02	1.23E-03
**ATM**	11	12.79	9.30	2.47E-04	6.85E-02	3.14E-02	4.82E-02	3.96E-02
**CASP1**	11	16.28	9.30	6.38E-04	1.57E-02	1.62E-04	2.52E-04	2.05E-07
**DDX10**	11	13.95	4.65	1.43E-03	1.77E-02	3.20E-03	4.26E-02	3.82E-10
**RAB30**	11	12.79	8.14	3.69E-03	1.06E-02	5.71E-03	5.27E-03	1.87E-06
**UBASH3B**	11	15.12	10.47	4.89E-04	8.16E-03	2.73E-03	1.75E-03	1.89E-03
**CLEC4A**	12	15.12	5.81	3.86E-03	3.56E-02	1.70E-06	4.61E-04	2.97E-07
**PPFIA2**	12	8.14	1.16	3.08E-03	4.92E-02	8.62E-04	4.11E-02	1.72E-04
**ENOX1**	13	12.79	12.79	3.28E-04	4.36E-02	2.50E-03	4.00E-02	1.76E-03
**PFAS**	17	18.60	13.95	2.93E-04	2.80E-02	1.35E-08	1.06E-02	1.65E-04
**PIK3R5**	17	20.93	13.95	1.36E-04	2.71E-02	1.89E-06	7.68E-03	1.96E-09
**SCO1**	17	26.74	11.63	4.86E-03	2.91E-02	9.85E-03	4.36E-02	3.64E-05
**BRD4**	19	16.28	8.14	3.65E-02	8.40E-03	1.82E-03	2.37E-03	2.27E-03
**CARM1**	19	18.60	2.33	4.43E-03	7.08E-03	3.84E-03	5.45E-03	2.04E-03
**IL27RA**	19	19.77	4.65	1.62E-03	8.35E-03	2.08E-03	1.09E-02	1.84E-03
**PODNL1**	19	18.60	4.65	3.81E-03	8.35E-03	1.35E-03	4.47E-02	8.86E-04
**CHAF1B**	21	15.12	6.98	1.16E-03	4.59E-02	6.07E-04	4.69E-04	3.96E-07

**PCNT**	21	16.28	11.63	5.31E-03	5.08E-02	3.86E-04	1.08E-06	3.20E-03

*_T as from training set; _V as from validation set.
